# Chemically Induced Colitis-Associated Cancer Models in Rodents for Pharmacological Modulation: A Systematic Review

**DOI:** 10.3390/jcm11102739

**Published:** 2022-05-12

**Authors:** Rita Modesto, João Estarreja, Inês Silva, João Rocha, Rui Pinto, Vanessa Mateus

**Affiliations:** 1 H&TRC—Health and Technology Research Center, ESTeSL—Escola Superior de Tecnologia da Saúde de Lisboa, Instituto Politécnico de Lisboa, 1990-096 Lisbon, Portugal; ritaemodesto@hotmail.com (R.M.); estarreja.20112014@gmail.com (J.E.); ines.silva@estesl.ipl.pt (I.S.); 2 iMed.ULisboa, Faculdade de Farmácia, Universidade de Lisboa, 1649-003 Lisbon, Portugal; jrocha@ff.ulisboa.pt (J.R.); rapinto@ff.ulisboa.pt (R.P.); 3 Joaquim Chaves Saúde, Joaquim Chaves Laboratório de Análises Clínicas, Miraflores, 1495-069 Algés, Portugal

**Keywords:** colitis-associated colorectal cancer, preclinical studies, disease animal models, animal experimentation, colorectal cancer

## Abstract

Animal models for colitis-associated colorectal cancer (CACC) represent an important tool to explore the mechanistic basis of cancer-related inflammation, providing important evidence that several inflammatory mediators play specific roles in the initiation and perpetuation of colitis and CACC. Although several original articles have been published describing the CACC model in rodents, there is no consensus about the induction method. This review aims to identify, summarize, compare, and discuss the chemical methods for the induction of CACC through the PRISMA methodology. Methods: We searched MEDLINE via the Pubmed platform for studies published through March 2021, using a highly sensitive search expression. The inclusion criteria were only original articles, articles where a chemically-induced animal model of CACC is described, preclinical studies in vivo with rodents, and articles published in English. Results: Chemically inducible models typically begin with the administration of a carcinogenic compound (as azoxymethane (AOM) or 1,2-dimethylhydrazine (DMH)), and inflammation is caused by repeated cycles of colitis-inducing agents (such as 2,4,6-trinitrobenzenesulfonic acid (TNBS) or dextran sulfate sodium (DSS)). The strains mostly used are C57BL/6 and Balb/c with 5–6 weeks. To characterize the preclinical model, the parameters more used include body weight, stool consistency and morbidity, inflammatory biomarkers such as tumor necrosis factor (TNF)-α, interleukin (IL)-6 and IL-1β, angiogenesis markers such as proliferating cell nuclear antigen (PCNA), marker of proliferation Ki-67, and caspase 3, the presence of ulcers, thickness or hyperemia in the colon, and histological evaluation of inflammation. Conclusion: The AOM administration seems to be important to the CACC induction method, since the carcinogenic effect is achieved with just one administration. DSS has been the more used inflammatory agent; however, the TNBS contribution should be more studied, since it allows a reliable, robust, and a highly reproducible animal model of intestinal inflammation.

## 1. Introduction

Colorectal cancer represents the third most diagnosed form of cancer, and it is a key cause of cancer mortality worldwide [1]. Indeed, this type of cancer is characterized by being responsible for 10% of all annually diagnosed cancer, and it is the world’s fourth most deadly cancer with, approximately 900,000 deaths annually [2]. Additionally, over 1.9 million new colorectal cancer cases and 935,173 deaths were predictable in 2020 [1,3], with more than half of the cases occurring in developed countries [4]. The disease prognosis is strongly related to the diagnosis stage; however, currently, there is a 5-year survival rate estimated at 66.1% for all stages of the disease [5,6].

Carcinogenesis of colon cancer departs from normal mucosa; however, through non-well-established steps, it tends to develop and leads to malignancy. It is a dynamic and multi-phase disease, which develops slowly over years and progresses through cytologically distinct benign and malignant states, from single crypt lesions through adenoma, to malignant carcinoma with possible metastasis [7]. In colorectal cancer, factors such as intestinal injury, oxidative stress, and chronic inflammation contribute to an alteration of the intestinal homeostasis [8,9,10].

Colorectal cancer pathogenesis could evolve from two different molecular pathways, sporadic and colitis-associated colorectal cancer. CACC results from defects in the cell cycle, even though different factors can be responsible for the neoplastic changes. Particularly, proinflammatory pathways, especially the NF-κB, IL-6/STAT3, COX-2/PGE2, and IL-23/Th17 signaling pathways, are dysregulated and consequently, they have a crucial role in the pathogenesis of CACC. The increased regulation of antiapoptotic proteins and the higher proliferation of epithelial cells, as well as new blood vessels, are essential in the tumor initiation, growth, and progression [7,8,9,10].

Inflammatory bowel disease (IBD) is characterized by a chronic inflammation of the large bowel including ulcerative colitis and Crohn’s disease and has been associated in several epidemiological studies with the spontaneous development of colorectal neoplasia, which is described as CACC. CACC is responsible for, approximately, 90% of the cases of CRC, which is allied with a possible hereditary influence [11,12,13,14,15]. Patients with IBD face an increased lifetime risk of developing CACC and a worse prognosis in comparison to healthy individuals [9,16]. Additionally, colon cancer is associated with significant morbidity and mortality up to 15% in IBD patients [17,18]. The risk for the development of CACC is closely associated with the extent of colonic involvement, duration of colitis, and severity of active inflammation [19,20,21]. The immune signaling pathways contribute to the pathogenesis of colitis and CAAC; however, only a limited number of studies were performed to understand the exact mechanisms underlying the link between chronic colitis and the development of CACC [9,22].

The treatment for CACC is personalized, taking into account the patient’s needs and tumor’s specificities [23]. In general, surgery is the mainstream curative treatment for CACC combined with radiotherapy and/or chemotherapy. However, several side effects are noticed due to the treatment toxicity. Side effects from chemotherapy for colon cancer include nausea, vomiting, loss of appetite, hair loss, mouth sores, diarrhea, and rashes. It also lowers white blood cell counts, so there is usually an increased risk of infection as well as low blood platelet counts resulting in bleeding or bruising. These treatments decrease life quality, and the survival rate depends primarily on the stage of the disease. Stage I has a 90% survival rate in 5 years against a 10% survival rate in stage IV in the same time spectrum [2,11,24].

Actually, numerous efforts have been made to find a new effective method to regulate undesirable immune responses during the autoimmune reaction [19]. The development of preclinical studies allows the evaluation of other therapeutic alternatives, knowledge of the pathogenesis, and possible future treatments, which are essential to improving the pharmacological approaches in the treatment of CACC [25]. Indeed, animal models represent an important tool to explore the mechanistic basis of cancer-related inflammation, where the induction of CACC can be perceived and manipulated by researchers [25,26]. Additionally, it also has an immense potential to provide important evidence about the inflammatory mediator’s roles in the initiation and perpetuation of IBD and CACC as well as in the development of new therapeutic approaches and their mechanism of action [7,25,26].

There are different types of animal models of CACC; however, chemically induced models are the greatest commonly used [25,27,28]. Such models are characterized by starting with the administration of a carcinogenic compound followed by repeated cycles of proinflammatory agents, which are toxic to colonic cells and generate an exacerbated inflammatory response [20,21]. This experimental intestinal carcinogenesis model should represent characteristics observed in human disease where tumors develop quickly and express biological modifications similar to those found in humans as well as mimic the disease progression from inflammation through dysplasia to carcinoma [29,30].

Currently, there is no agreement in the literature concerning the induction method taking into account several parameters, such as doses and/or concentrations of the carcinogenic and proinflammatory agents, frequency of administrations, gender, age, and strains of the mice utilized. Thus, our research group purposes to identify, summarize, compare, and discuss different protocols for the induction of CACC through the PRISMA methodology. The challenge of the present systematic review is to promote a synthesis of the information available in the literature, which can be an important tool for future research on new possible pharmacological approaches for the treatment of CACC, contributing to a more effective and safe treatment.

## 2. Materials and Methods

### 2.1. Search Strategy 

**Figure 1:**
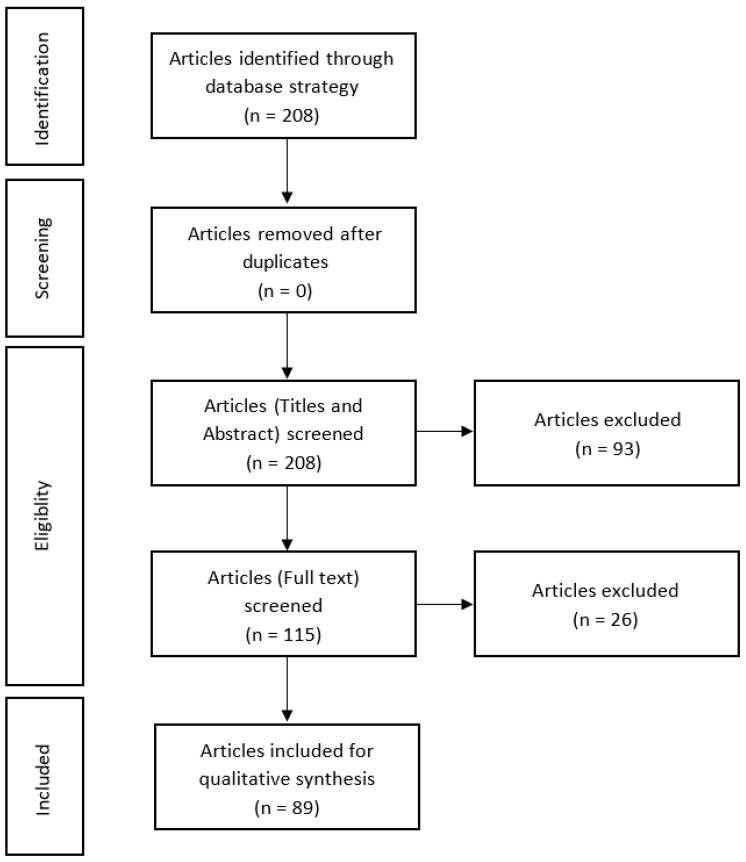
PRISMA flow diagram showing results of the literature search.

Following the establishment of a review protocol based on PRISMA methodology, the electronic database MEDLINE via the PubMed platform was searched from initiation up to March 2021 for all studies with a chemically induced animal model of CACC in mice. The search strategy was initiated with the insertion of keywords in the MeSH Database in order to find MeSH terms. Posteriorly, a combination of the keywords was carried out, and the final search expression was: (“Mice”[Mesh] OR Mice[tiab] OR Mice OR Mouse) AND (“Animal Experimentation”[Mesh] OR “Animal Experimentation”[tiab] OR “Animal Experimentation” OR “Preclinical studies”[tiab] OR “Preclinical studies” OR “Non-clinical studies”[tiab] OR “Non-clinical studies” OR “animal model”[tiab] OR “animal model” OR “disease models, animal” [Mesh] OR “disease model”[tiab] OR “disease model” OR “disease models”[tiab] OR “disease models” OR “disease animal model”[tiab] OR “disease animal model” OR “disease animal models”[tiab] OR “disease animal models”)) AND (“colitis-associated neoplasms”[Mesh] OR “colitis-associated neoplasms”[tiab] OR “colitis-associated neoplasms” OR “colitis-associated colorectal cancer”[tiab] OR “colitis-associated colorectal cancer” OR “colitis-associated cancer”[tiab] OR “colitis-associated cancer”). The results of the literature search are outlined in Figure 1.

### 2.2. Selection of Studies

In order to select the articles after the search expression was performed, there were several inclusion criteria taken into account, such as: (1) only original articles; (2) articles where a chemically induced animal model of CACC is described; (3) preclinical studies in vivo with rodents; and (4) articles published in English. Additionally, the research group also selected exclusion criteria, such as: (1) review articles; (2) short communications; (3) case reports, and (4) expert opinions. Throughout the process of selecting the studies, the exclusion started with the analysis of the abstracts, and then, the full texts of the remaining articles were retrieved and reviewed. In case of disagreements between the reviewers and the absence of consensus, a third element was included to make the final decision. 

### 2.3. Data Extraction

The data of interest were independently extracted by both reviewers into a Microsoft Excel spreadsheet (Windows 10 edition; Microsoft Corporation, Lisbon, Portugal). As well as in the process of selecting the articles, the possible disagreements between the reviewers were resolved by mutual consensus or by the inclusion of a third element to make the final decision. The information of interest extracted from each study was as follows: pro-inflammatory reagent-related parameters (number of administrations, frequency of administration, doses, volume, concentrations, and vehicles used), procarcinogen reagent-related parameters (number of administrations and doses), mice-related parameters (strain, gender, and animal age), model characterization (clinical signs and symptoms, biochemical markers and inflammatory and angiogenesis biomarkers, macroscopic evaluation, and histological evaluation), authors and year of publication.

### 2.4. Articles Eligibility

Related to our article’s eligibility, studies using a carcinogenic chemical combined with a proinflammatory substance were selected, promoting the development of tumors with an inflammatory response associated. The parameters such as dosage, timing, and frequency of administration were all included, since the objective is to compare and evaluate their influence in the induction method. We excluded the utilization of only a procarcinogen agent without a proinflammatory chemical, which mimics the development of cancer in the colon without an inflammatory response associated. Studies with genetically modified strains of mice were also excluded because tumors develop in the colon without the action of chemicals. Finally, studies with transplanted tumors were excluded, too.

After performing the search strategy, the retrieved articles were exported from the MEDLINE database to a Systematic Reviews Web Application (Rayyan QCRI), and the titles and abstracts were analyzed by two independent reviewers in order to select the relevant and potentially eligible studies. Then, after selecting all the articles, the same two independent reviewers assessed the full text of each one and decided whether the article was eligible or not, considering the inclusion and exclusion criteria. In these two steps, a third element was included in case of discrepancies between the two reviewers in order to provide a final decision. After the selection of the eligible studies, the same two independent reviewers extracted the relevant data present in those and inserted them in a customized data extraction document. The data of interest were extracted from the text, graphs, and/or tables present in the chosen articles. In case of discrepancies and an absence of a consensus at the end of the task, a third element was included in order to make a final decision. 

To evaluate the internal validity of the selected studies and the methodological quality, through the analysis of the potential risk of bias present, we used SYRCLE’s risk of bias tool. Throughout the process, several key points will be formulated to be aware of the analysis of each study and attributed a punctuation, which in the end culminated in a final score for each article.

Our animal systematic review was posteriorly submitted to PROSPERO, which is an international prospective register of systematic reviews.

Animal care was in accordance with the internationally accepted principles for laboratory animal use and care, Directive 2010/63/EU.

Thus, this review aims to identify, summarize, and analyze different chemical methods for inducing colitis-associated cancer in mice for that we propose to compare and debate some important parameters, such as proinflammatory reagent-related parameters, procarcinogen reagent-related parameters, mice-related parameters, and the model characterization.

## 3. Results

The electronic database allowed identifying 208 publications in total after the application of the search expression. The publications were then screened according to the inclusion and exclusion criteria (Figure 1). No duplicates were identified in the abstract analysis; however, 93 original articles were excluded, since they were published more than 5 years ago. Of these, 115 published articles appeared to be pertinent to the study question and were saved for extra assessment. From the 115 papers remaining, 25 were not aligned with the purpose of the work and were excluded as ineligible based on the inclusion criteria described previously. The reasons for the excluded articles were: the article corresponds to a protocol (*n* = 4); letter (*n* = 1); short communication (*n* = 1); review (*n* = 2); systematic review (*n* = 1); method of induction that does not use only chemicals (*n* = 10); tumour induction at other organs (*n* = 1); articles where a chemically induced animal model of CACC was not described (*n* = 3), and the article was written in Chinese (*n* = 2). Thus, 89 original articles were included in the qualitative analysis, since all of these studies have described a chemically induced animal model of CACC in rodents (Table 1).

Preclinical studies in vivo, particularly animal model studies, mimic the pathogenesis of CACC disease in humans and allow testing new pharmacological approaches, and they are vital for knowing the underlying pathogenesis and for conceivable upcoming treatments [31,32]. There are several types of animal models of CACC: chemically induced models; transplantation models, those that express intestinal inflammation spontaneously, those in which intestinal inflammation can be induced by specific immunological methods, the genetically engineered models by gene knockout, knockin, or transgenic methods, and the last includes adaptative transfer models [33,34]. The chemically induced models are studied in the greatest detail so far for CRC. The CACC models are appropriated to develop and test novel therapeutic strategies for the treatment of the disease. The knowledge of molecular pathways involved in CACC may provide opportunities for innovative therapeutic strategies for the treatment of CACC in the future. The exact involvement of genetic susceptibility, microenvironment, and immune reactivity remains unclear; therefore, the prevention and therapy of CACC are challenging [35]. There is interest in the use and study of more than one animal model, since differences between models may reflect the different subgroups of patients with IBD. The most used chemicals to induce colitis models are 2,4,6-trinitrobenzenesulfonic acid (TNBS), which promotes a Th1 response, resembling CD, and dextran sulfate sodium (DSS), which promotes a Th2 response, resembling UC [36,37]. DSS-induced colitis and TNBS-induced colitis models are the most widely used to induce IBD since they symptomatically, morphologically, and histopathologically resemble human IBD and allow the development and test of novel therapeutic strategies [38,39,40]. 

DSS is easy to use and briefly obtain results resembling UC in humans [41,42]. To cause inflammation in rats or mice, DSS protocol uses DSS added to drinking water; then, acute or chronic colitis model experiments can be conducted only by altering the concentrations of the administered substance as well as the number of cycles of supply of the chemical agent. The severity of colitis caused by DSS depends on the dose, duration of administration, and animal strain [38,39] as well as the manufacturer and molecular weight of DSS, gender, and animals raising environment such as germ-free or specific pathogen-free environments [43,44].

The TNBS model is an easily induced, rapid, reliable, robust, and highly reproducible animal model of intestinal inflammation. The induction of the disease occurs quickly and appears 4 to 7 days after intrarectal administration of the TNBS hapten reagent, gradually progressing into a chronic pattern during at most approximately about 8 weeks [45,46,47]. Protocols of the chronic TNBS-induced colitis model are not standardized concerning the dose of TNBS, the depth of TNBS administration, the animal strain, and the time point for model evaluation [48].

The CACC animal model requires first the administration of a procarcinogen compound AOM or DMH. AOM is used to enhance the formation of colorectal tumors. AOM is transported to the liver and is metabolized by cytochrome P450 to methylazocymethanol, which is a highly reactive alkylating species that induces nucleotide transitions, the active agent which is then secreted with bile into colonic epithelium, inducing mutagenesis. DMH is also a compound used experimentally to induce tumors in animal models of carcinogenesis, since it induces carcinogenesis through deregulation of the cell cycle, acting as a DNA methylating agent [49,50]. 

This experimental animal model of CACC must be capable of providing an intestinal carcinogenesis model, where tumors develop over the short term and express biological modifications similar to those found in humans. The two-step tumor model of CACC mimics the progression of CACC development in humans from inflammation through dysplasia to carcinoma [48,49,50].

However, a consensus in the CACC procedures model is not achieved, resulting in the absence of a standardized protocol for the development of the disease. Parameters such as the doses and concentration of proinflammatory agent, procarcinogen agent concentration, the animal strain, and the time point for model evaluation remain indefinite, creating a deficiency in obtaining a reproducible model [26,50]. Since there are several accessible CACC models, the main concern of the researchers is having all of the variables, previously referred, to in deliberation for future application in the preclinical testing to achieve the greatest possible results [35].

### 3.1. Pro-Inflammatory Reagent Related Parameters 

#### 3.1.1. Number of TNBS Administrations 

TNBS induces an acute and chronic form of colitis dependent on the dose and frequency of administration, reacting with some amino acid groups on the intestinal mucosa and bacterial proteins of the colon and rendering them immunogenic. This model is based on increased permeability of the membrane that occurs in IBD, which facilitates the entry of a luminal antigen that is not adequately eliminated by the immune system, the haptenization [140,141]. A large part of the articles consider that the chronic colitis only can be induced by more than one administration; however, our data only find one article with TNBS, with one single administration, indicating a bigger use of DSS in CACC mouse models in the last 5 years, allowing the opening of a new window of knowledge with the use of TNBS as a disease inducer. The main advantages of this model include a simple and low-cost protocol and reproducible colonic damage, short experiment duration, enduring damage accompanied by inflammatory cell infiltration, and ulcers. The know-how of our research group in the development of the chronic mouse model of colitis as well as the literature defending those repeated administrations of TNBS are preferred, resulting in a local Th1 response that has the characteristics of Crohn’s disease. Other authors refer to a dose-escalating or repeated enemas of TNBS as a possible strategy to achieve the induction of chronic colitis, but never by oral feeding, since this will endorse significant oral tolerance [142,143]. Built from the knowledge of our research group in the development and validation of a chronic mouse model of colitis, TNBS-induced chronic colitis should be developed in 4 weeks, providing a chronic intestinal inflammation model. Accordingly, the acute transmural damage became maximal from 3 days to 1 week after instillation and resolved within 2 weeks; however, if multiple TNBS administrations are used, the colonic inflammation can gradually progress, lasting for about 8 weeks [34,144,145]. In addition, the disease severity and clinical course may be altered with the use of a TNBS hapten suboptimal reagent [48]. 

#### 3.1.2. TNBS Dose 

To generate chronic colitis, the optimization of TNBS concentrations is important. The dose to induce colitis oscillates due to several key factors, including genetic background, gender, age, body weight, as well as sterility conditions of the animal facility and strain. According to the literature, adjusting the respective doses of TNBS may bring about a spectrum of disease, from acute to chronic. While high dosages of TNBS lead to massive colitis, necrosis, colon perforation, and consequently an acute mortality rate due to massive colitis, lower dosage may be inefficient in the induction of colitis, causing short-lasting, weak, or even completely absent disease activity [32,45,146]. Relatively to TNBS dose, our data refer to a 2.5 mg single dose; however, based on previous studies about chronic colitis from our research group, doses ranges can vary from 0.3 to 5.0 mg per mouse (for an average body weight in each mouse of 20 mg) to induce chronic colitis [30]. All experimental studies should be performed in a selected area exclusively for the colitis induction with TNBS, and the sterility conditions of the animal accommodations should be assured, as they have a large impact on disease outcome. In addition, the cohabitation of the experimental mice with other strains of mice or pathogens may modify the immune response and consequently the expected results [37].

#### 3.1.3. TNBS Volume 

As well as the dose, the volume of administration is a crucial parameter to evaluate by the investigators before administering any substance to an animal. The recommended volumes of administration are described in guidelines, considering the route of administration, the toxicity of the administrated substance, and the size of the rodent. Inappropriate volumes of solution can shock the animal. Rectal administration is an enteral administration made directly in the gastrointestinal tract that can be performed using soft small-gauge flexible tubing with a dosing syringe attached to the end. In the mouse, the injection volume limit on rectal administration is 500 µL [147].

In this review, the authors used enemas with 150 µL, which are in agreement with the literature. According to our experience with rectal administration in mice, the injected volume varies between 50 and 500 µL; nevertheless, the risk of leakage is higher for volumes above 100 µL [30]. However, there is no consensus about any recommend ideal volume for rectal administration in mice. To prevent colonic reflux, the mice should be post-maintained in the Trendelenburg position after the rectal administration, since a lack of practice in the technique, presence of feces in the colon, anatomical positioning of the descending colon, and injection rate of the volume to be administered can contribute to the rectal reflux of TNBS and consequently promote deficiencies in the induction method or increase the variability in animals’ disease.

These findings suggest that there is no defined volume. It is desirable always to use reduced volume to the same dose in order to ensure the complete absorption and retention of the entire solution to reduce the commitment of a correct validation model.

#### 3.1.4. TNBS Vehicle 

The range known of ethanol concentrations used in the literature varies between 10 and 80%; however, most studies use ethanol between 45% and 55% as a TNBS vehicle, accordingly to the optimal dosage of ethanol described as 30% to 50% [41,45]. The ethanol permeabilizes the epithelial layer that separates the luminal contents of the colon from the cells of the mucosal immune system, allowing the penetration of TNBS in the bowel wall, the ethanol at 50% is the most recommended to disrupt the intestinal barrier and enable the translocation of the TNBS into the submucosal layer. Still, some authors use lower concentrations of ethanol in order to avoid ethanol interference in inducing damage to the intestinal epithelium, but there is no described evidence of the effects of 50% ethanol in colon lesions in the TNBS colitis model. In our previous chronic colitis model, the findings corroborate the same [148,149,150,151,152,153]. The use of ethanol is only required to break the intestinal barrier, increasing its permeability [25,30,154,155]. 

#### 3.1.5. Number of DSS Administrations 

DSS-induced colitis is a reproducible model that morphologically and symptomatically resembles UC in humans. DSS acts as a toxin to colonic epithelium originating epithelial cell injury, the disruption of the intestinal epithelial monolayer lining outcomes in colonic inflammation, resulting in the entrance of antigens and luminal bacteria in the bowel mucosa, permitting the exacerbation of the inflammation and spread of the intestinal contents into the tissue [141]. The commonly used protocol for DSS-induced colitis in mice is to add DSS to drinking water in a dose range of 2–10% by repeated exposure administering in three to five cycles punctuated with recovery periods. The addition of DSS to drinking water, modifying the concentration of DSS, and the frequency of administration permit obtaining a very reproducible acute or chronic and relapsing model of intestinal colonic inflammation as well as a useful model for a better understanding of the innate immune mechanisms of UC [144,145]. The severity of the DSS-induced colitis model depends on the dose, duration of administration, and animal strain. However, the DSS model presents some disadvantages, such as the cost and the possible variations in disease severity, taking into account the presence of impurities in the DSS preparation or the quantity consumed by each mouse. In addition, the disease is characterized by progressive crypt dropout, suggesting a direct effect of DSS on the epithelial cells as opposed to lamina propria cells as suggested in human IBD [144,145,146]. 

Attending our studied papers, we observe an extensive number of papers using DSS as an inducer of colitis in the studied animal models (*n* = 89) just like a wide range of administration patterns. The administration design varies according to the different authors from one DSS cycle to four DSS cycles of 5 or 7 days followed by a recovering period. The majority of our papers indicate three DSS cycles as preferable (*n* = 41) contrarily to one DSS cycle (*n* = 7) or four DSS cycles (*n* = 3), leading us to believe that fewer DSS cycles should not be sufficient to establish the disease or even develop a chronic pattern, and similarly, plentiful DSS cycles could be prejudicial to the animal, taking into account his life span and posteriorly lead to death.

#### 3.1.6. DSS Dose

DSS is usually administered in a dose range of 2–10% for 5–10 days to induce acute inflammation following a single continuous exposure. By prolonging DSS administration, acute colitis may be extrapolated to chronic colitis by repeated exposure administered in three to five cycles interrupted with recovery periods [144,145,146]. 

In our review, we could find dose variation between 1% and 3% of DSS dose. The great part of the analyzed papers maintains the initial dose during the treatment; however, some authors refer to the use of increasing doses during the treatment to obtain a pre-sensitization effect, which permits reducing the mortality rate with higher doses, and, according to the authors, obtaining a more reliable chronicity animal model. The most applied dose is 2% DSS observed in thirty-nine of our studied articles (*n* = 39); however, we observe a great fraction of the articles using 3% DSS in the animal models (*n* = 15). Meanwhile, only two articles refer to the use of 1% of DSS as enough to develop a chronic model of colitis to induce CACC (*n* = 2). However, DSS promotes a generalized inflammation in the whole intestine, including the colon and rectum, which allows us to assume TNBS as a better chemical inducer in an animal model of CACC.

### 3.2. Procarcinogen Reagent Related Parameters 

#### 3.2.1. Number of AOM Administrations

A procarcinogen is a compound that is not itself carcinogenic but undergoes metabolic activation in the body to yield a carcinogen. AOM is a metabolite of the procarcinogenic 1,2-dimethylhydrazine (DMH). The mutagenic agent AOM initially needs metabolic activation to form DNA-reactive products. Firstly, in the liver cytochrome, P-450 isoform hydroxylates AOM to the stable methylazoxymethanol glycoside (MAM), which is then transported to the colon where it finally promotes DNA damage. In our review, the articles are completely elucidative with an overwhelming majority of the papers using AOM as a procarcinogen (*n* = 89). As described in the literature, AOM on par with 1,2-dimethylhydrazine (DMH) are the most used procarcinogens in mice models. DMH, a metabolic precursor of MAM, was used in several early studies to induce tumors in rats [11,13,17]. Repetitive treatment with this methylating agent was reported to produce colon tumors in rodents that exhibit many of the pathological features associated with the human disease [19,20,21,22]. Thus, DMH has provided cancer researchers with a reproducible experimental system for studying forms of CACC. However, AOM offers advantages over DMH, including enhanced potency and greater stability in dosing solution [16,18,22]. The number of procarcinogen administrations was also targeted by our review; the majority of the articles indicate preferably the use of only one single administration of AOM (*n* = 56), others use two administrations (*n* = 5), and the last author uses five administrations of the procarcinogen AOM (*n* = 1). The AOM administration normally is performed one week before the following treatment. The induction as well as the number of administrations has a crucial role in the AOM absorption by the animal organism and consequent carcinogenesis model success. The data demonstrate a preference for the intraperitoneal administration of AOM (*n* = 84) against one paper that describes the administration intravenously; besides this, intrarectal administration is well described in the literature. However, the performance of carcinogen administration should be preferably intraperitoneal.

#### 3.2.2. AOM Dose

A wide range of azoxymethane concentrations can be identified from our selected articles; the lowest dose observed was 3 mg/kg, and the highest dose was 20 mg/kg. Nevertheless, we observed a consensus in the majority of the CACC inductions with an optimal concentration of 10 mg/kg (*n* = 53). In the literature, some authors perform a dose–response study with AOM and different doses of a proinflammatory agent, suggesting that severe types of inflammation and nitrosative stress were caused by high doses of the proinflammatory agent. Thus, the tumor-promoting effect is dose-dependent, and the effect corresponds to the degree of inflammation and nitrosative stress, which is assessed in this study by an increased variety of cell types (neoplastic, cryptal, and endothelial cells, as with infiltrative mononuclear cells) within the colonic mucosa [22].

### 3.3. Rodent-Related Parameters 

#### 3.3.1. Strain 

In the animal models, the susceptibility to develop the studied disease varies with strains; hence, it is important to choose the correct animal model strain. Preclinical studies of experimental colitis have been developed in different animal species such as rats, mice, pigs, rabbits, nonhuman primates, and dogs [156,157]. The majority of the animal models using mice demonstrate susceptibility to disease development at a rate of 90% [45]. Originally, SJL/J mice were described as the mouse strain with higher susceptibility for the induction of colitis [145]; actually, this fact remains well accepted in actual scientific data [32,158]. Mainly in DSS animal models, C3H/HeJ and Balb/c mice strains are pronounced as more susceptible [146]. However, strains such as BALB/C and C57BL/6 are frequently used with success for the development of colitis with different inflammatory agents. The comparison between strains demonstrates a relative resistance between Balb/c mice and C57BL/6, and SJL/J, which can be mitigated with the use of pre-sensitization; the data are not shown in our review. According to our data, the most frequently used strains to induce chronic colitis were C57BL/6 (*n* = 59) and Balb/c mice (*n* = 16). It is important to ponder that the inconsistency between mice strains requires also the optimization of the proinflammatory reagent concentration [41], as we have mentioned before.

#### 3.3.2. Gender 

Relatively to animal gender, our analyzed studies allow us to understand that there is no tendency toward gender. There are several articles referring to the use of males and females in the same protocol (*n* = 11). A great percentage of the papers use males (*n* = 39) against an inferior number using females (*n* = 17). However, some articles do not even refer to gender (*n* = 15). There is no agreement about this topic in the literature, since some studies argue for a more exacerbated disease in males, and on the other hand, some authors studied particularly the hormonal involvement in the disease model progression, concluding no association between the gender and the disease evolution. Particularly, in DSS animal models, gender can influence the severity and susceptibility of exposure, since some authors describe males as more susceptible to developing the disease [144]. Nevertheless, the literature indicates that both males and females can develop an animal model with the same clinical characteristics [45]. Thus, it seems that there is no variation in the results about the gender. 

#### 3.3.3. Age 

According to the literature, animal age represents a significant parameter in animal model studies, since it is directly related to the animal susceptibility to the disease and the consequent mortality rate. The data from our investigated articles demonstrate a large range of ages, specifically between four and fourteen weeks. However, some studies do not mention the animal age (*n* = 10). Scheiffele and Fuss described colitis induction with animals at 5 to 6 weeks of age, because younger animals have a greater success rate of induction; otherwise, animals up to 4 weeks of age suffer an increased mortality rate [45]. Instead of age, other articles define the weight instead the age of the animal or even combine both. The used average weight is around 20 g (data not shown), which conforms to the weight of an adult mouse [147]. Regarding this parameter, it is being considered that colitis induction should be performed in animals between 18 and 20 g of body weight. Nevertheless, the importance retained from the data is the preferential use of adult mice in preclinical studies.

### 3.4. Model Characterization

The evaluated papers are coherent in the majority of parameters analyzed. The authors evaluate clinical signs and symptoms, biochemical markers, and observed macroscopic lesions, and then make a histological assessment of the colon samples. Still, different parameters are investigated in different papers considering the preclinical models proposed. Summarily, we describe each one of them below. 

#### 3.4.1. Clinical Signs and Symptoms 

During the experimental development, the animals were observed daily, and we monitored clinical signs throughout the evaluation of different parameters, such as body weight, morbidity, stool consistency, and anus appearance. Regarding scrutinized articles, we expected alterations of intestinal motility characterized by diarrhea or soft stools, edema of the anus, and moderate morbidity, accompanied by a general deterioration in their appearance. The studied groups presented a decrease in body weight, demonstrating that sick mice became weaker, with progressive weight loss and subsequently increased mortality. These are in agreement with other authors who describe that the animals develop visible signs of disease and decrease in the activity level [33,45]. Although the clinical signs and symptoms appear to be fewer sensitive parameters (especially body weight and morbidity), almost all of the observed studies with this preclinical model have shown the necessity of monitoring them (*n* = 63). However, in addition to the aforementioned signs/symptoms regularly evaluated, it is suggested that the principles of the Rat/Mouse Grimace Scale should always be monitored daily, as they allow for better monitoring of pain and, thus, a better perception/assessment of model evolution. Some criteria are the closure of the orbital area, the protuberance of the nose when the animal is not under active exploration, the contraction of the cheek muscle, and the position of the ears and whiskers.

#### 3.4.2. Biochemical Markers 

The severity and the occurrence of pathology can be detected or measured in blood or tissues through biochemical markers. From our collected data, we find a significant percentage of articles using the determination of biochemical markers, since it allows a precise parameters quantification in blood to determine the severity of colitis [159,160,161]. (*n* = 16). In the literature, we can find that the analysis of serum is conducted in order to evaluate several parameters, such as alkaline phosphatase (ALP) and extra-intestinal manifestations, urea, creatinine and alanine aminotransferase (ALT), aspartate aminotransferase (AST), albumin as well as additional analyses, such as fecal hemoglobin. ALP is expressed by the intestinal epithelium; it has an important role in mucosal defense and will be determined as a marker of intestinal homeostasis. Extra-intestinal manifestations are evaluated as representative of external and consequent manifestations of the inflammation. All of them are evaluated spectrophotometrically. Urea, creatinine, and alanine aminotransferase (ALT) are biomarkers not significantly evaluated by the articles studied in this review (*n* = 2). However, based on the know-how of our research group in the anterior developed mouse model of TNBS-induced colitis, these parameters allow the evaluation of the extra-intestinal influence of our induction model. These biochemical markers, non-related directly to the intestine, are representative of external and consequent manifestations of the inflammation. Urea and creatinine are determined as markers of renal function, and ALT is determined to be a marker of hepatic function. Consistent with the literature, the higher serum levels represent extraintestinal manifestations and secondary effects involved with almost every organ system, and some of the most frequently involved organs are the liver and kidney [162,163,164,165]. TNBS-induced colitis is therefore expected to show a significant change in renal and hepatic functions compared to the control groups, which is characterized by increased levels of these markers in serum. Feces are collected from all groups in order to measure the fecal hemoglobin. Fecal hemoglobin is evaluated using a quantitative method by immunoturbidimetry as an index of hemorrhagic focus. Our research group used fecal hemoglobin, an extremely sensitive parameter, in the acute colitis model. The determination of fecal hemoglobin allows the diagnosis and evaluation of various colorectal diseases once it determines the intensity of the hemorrhagic focus in the damage of colonic tissue [162,163,164,165]. In this sense, we expect to have high values of fecal hemoglobin in the colitis groups as opposed to the control group, where we suppose that they present residual fecal hemoglobin concentrations. Fecal hemoglobin is determined in our data in ten articles (*n* = 10).

#### 3.4.3. Pro-Inflammatory Markers

In the analyzed articles, several distinct inflammatory biomarkers are described as interferon (IFN)-γ (*n* = 13), tumor necrosis factor (TNF)-α (*n* = 38), myeloperoxidase (MPO) (*n* = 10), cyclo-oxygenase-2 (COX-2) (*n* = 21), interleukin (IL)-6 (*n* = 42), IL-12 (*n* = 4), IL-1β (*n* = 21), and IL-10 (*n* = 18). Cytokines are molecules involved in signal emission between cells during the triggering of immune responses and are crucial for fighting infections and other immune responses. The proinflammatory cytokines, IFN-γ, TNF-α, IL-6, IL-12, IL-1β, and the anti-inflammatory cytokine, IL-10, are the most used as biomarkers and should be measured in the colon with a spectrophotometer. Proinflammatory cytokines work by promoting the inflammatory process, ensuring that reactions occur and consequently the initial insult is eliminated. In immune responses, IFN-γ, TNF-α, IL-6, IL-12, and IL-1β are proinflammatory cytokines released after triggering the inflammatory process. The increased values of these proinflammatory cytokines are related to IBD pathogenesis once they are augmented in colitic tissue [166,167]. TNF-α is a proinflammatory cytokine produced during the innate immune response of IBD. TNF-α is associated with the pathogenesis of colitis since is increased in inflamed colon tissue. Anti-inflammatory cytokines act as a brake on this process, preventing an exacerbated response and possibly producing undesirable effects of the inflammation itself and the healing process [166,168]. The presence of anti-inflammatory cytokines, such as IL-10, suggests a decreased serum value in different data, which is consistent with the expected hypothesis that the immune system in the presence of a chronic inflammatory insult tends to dispel the disease, balance values of biochemical markers of inflammation, and consequently re-establish homeostasis [169,170]. IL-10 plays a central role in the mucosal immune system by inhibiting proinflammatory cytokine synthesis and antigen presentation, and at the same time, it relieves intestinal inflammation [171]. COX-2 is another biomarker widely found in our data; it is an enzyme responsible for the phenomena of inflammation and production of prostaglandins, which is largely studied in the literature of the inflammatory process. MPO is a peroxidase enzyme most abundantly expressed in neutrophil granulocytes. The MPO activity is indirectly related to neutrophil infiltration in the inflamed colon. In the presence of inflammation, the MPO enzyme is released from the colonic mucosa, allowing a direct correlation of its release with the values in the systemic circulation [94]. The specific inflammation markers studied in the colitic mouse models represent and prove the consistency of the induction method used as well as confirm the occurrence of inflammation in the intestine caused by colonic damage. 

#### 3.4.4. Carcinogenesis Markers 

Angiogenesis is essential for tumor growth and metastatic spread. Therefore, angiogenic factors are important targets of anti-tumor therapy. Samples collected from colonic tissue should be used for spectrophotometric measurement in a spectrophotometer to determine tumor markers levels [155]. Markers such as vascular endothelial growth factor A (VEGF-A), granulocyte colony-stimulating factor (G-CSF), chemokine (C-X-C motif) ligand 1 (CXCL1), β- catenin, proliferating cell nuclear antigen (PCNA), a marker of proliferation Ki-67, caspase 3 and epidermal growth factor (EGF) are found in the literature as well as are present in our collected data, and they are important as representative of tumor progression. VEGF-A is a signal protein produced by many cells that stimulates the formation of new blood vessels and angiogenesis (*n* = 7). GCSF is a cytokine that stimulates the production of granulocytes and stem cells (*n* = 8). CXCL1 has a role in angiogenesis and thus has been shown to act in the process of tumor progression (*n* = 10). β-catenin plays a role in the most diverse pathways of cell signaling, acting mainly as a transcription factor, which mainly highlights its essential signaling in developmental biology and as a protein involved in cell adhesion (*n* = 11). Mutations and super expression are associated with various types of cancer, including pulmonary, breast, ovarian, endometrial and hepatocellular, and colorectal carcinomas. Proliferating cell nuclear antigen (PCNA) was originally identified as an antigen that is expressed in the nuclei of cells during the DNA synthesis phase of the cell cycle (*n* = 16). Ki-67 is an excellent marker to determine the growth fraction of a given cell population (*n* = 21). The fraction of Ki-67-positive tumor cells is often correlated with the clinical course of cancer. Antibodies against PCNA or monoclonal antibody termed Ki-67 can be used for grading different neoplasms. They can be of diagnostic and prognostic value. The imaging of the nuclear distribution of PCNA can be used to distinguish between the early, mid, and late S phases of the cell cycle. The caspase 3 protein by his side plays a central role in the execution phase of cell apoptosis (*n* = 13). Lastly, EGF is important for cancer cell proliferation, angiogenesis, and metastasis in many types of cancer [155,172,173]. There is a broad angiogenesis marker that represents key molecules in angiogenesis and vasculogenesis; however, its molecular mechanisms of action remain incompletely understood with no discussion about its importance in the evaluation of the disease progression.

#### 3.4.5. Macroscopic Evaluation 

Relatively to the macroscopic evaluation, colons must be observed macroscopically and scored to gross morphology according to the Morris method, and several parameters can be analyzed in the necropsied colon, such as weight and length of the colon, wall thickness, hyperemia, ulceration, and adhesions. The macroscopic evaluation, although unspecific, represents an important tool in the disease characterization, as present in our data (*n* = 29). In inflammation, the gut wall tends to increase in the thickness, and the macroscopic observation of the bowel also demonstrates ulcerations and hyperemia, which are parameters analyzed in data that are consistent with those expected by other authors and the presence of disease. Macroscopic score evaluation of the colon was based on descriptions by Morris et al. (1989) [145]. Colon length (*n* = 28) is evaluated in our studies as a picture of intestinal damage and measured as a marker of tissue integrity, which is determined using a measuring scale; the literature points to a reduction in colonic length after TNBS treatment, indicating a reduction in the colon length after the disease installation [174,175,176,177]. 

The number, incidence, size, and distribution of tumors (*n* = 79) were the most scrutinized parameters observed macroscopically, which represents the capability of the induction method in developing polyps as well as the location in the gut and dimensions.

#### 3.4.6. Histological Evaluation 

Histopathology is carried out by two independent histopathologists blinded to the treatment groups. The colon samples are fixed, processed routinely for paraffin embedding, sectioned, and stained with hematoxylin and eosin. The assessment of colitis-associated cancer is scored based on the sum of the inflammation score and carcinogenesis score of colons. The histopathological score of lesions is partially scored with some parameters: namely, the presence of tissue loss/necrosis, the severity of the mucosal epithelial lesion, inflammation, the percentage of intestine affected in any manner, and the percentage of intestine affected by the most severe lesion. The articles show histological evaluation as an important parameter to be considered, since it is present in several examined papers (*n* = 30). The histologic analysis method allows a qualitative evaluation of the analyzed sample, since it determines the impact in colon tissue, the change in intestinal permeability, and the damage in the colon tissue as well as the inflammation severity in the intestine [178,179,180,181].

## 4. Conclusions

In CACC research, preclinical studies in vivo are pillars for understanding the pathogenesis and possible future treatments. Murine models have an immense potential, where the induction of CACC can be witnessed and manipulated by researchers [24]. The results from animal models research may provide insight into potential therapeutic approaches to ameliorate the inflammation and minimize the morbidity and mortality associated with CACC. However, variability in the results in preclinical studies, due to several conditions such as type of induction method, administered doses, and treatment period, makes the translation of the data for clinical practice difficult. Careful attention may be required to translate animal studies to clinical settings by ensuring that both safety and efficacy can be modeled [2]. 

Based on this review study, the previous AOM administration seems important to the CACC induction method; however, just one administration is necessary to stimulate the carcinogenic effect. DSS has been the more used inflammatory agent to promote chronic colitis. However, more studies with the TNBS contribution are suggested, since this inflammatory agent allows a reliable, robust, and highly reproducible animal model of intestinal inflammation.

The strains mostly used are Balb/c and C57BL/6 with 5–6 weeks with males or females. The most used parameters to characterize this preclinical model include clinical signs and symptoms (body weight, stool consistency, and morbidity), the concentration of inflammatory biomarkers (IFN-γ, MPO, TNF-α, IL-6, and IL-10), the concentration of angiogenesis markers (VEGF-A, G-CSF, CXCL1, β-catenine, PCNA, Ki-67, caspase 3 and EGF), macroscopic evaluation of the colon (ulcers, thickness, and hyperemia) and histological evaluation of the colon. Since the two-step tumor model of CACC mimics the progression of CACC development in humans from inflammation through dysplasia to carcinoma, this systematic review allows us to better understand the different methods to induce a CACC model. Considering that there are several protocols published for inducing this disease in animals through the use of chemicals with the absence of a standard process, this systematic review summarizes and analyzes the different chemical procedures existent and the underlying evidence. In this sense, this systematic review provides a clearer vision about the use of chemicals in the development of CAC in animal models, which can be useful for the scientific community in terms of the formulation of a protocol based on the information present in the final version of this study.

## Figures and Tables

**Table 1:** Summarized outcomes of induction methods for CACC models.

Procarcinogen Reagent-Related Parameters—AOM		Pro-Inflammatory Reagent-Related Parameters						Rodent-Related Parameters			Model Characterization	Reference
Number of Administrations	Dose (mg/Kg)	Number of TNBS Administrations	TNBS Dose (mg)	TNBS Volume (µL)	TNBS Vehicle	Number of DSS Administrations (Cycles)	DSS Dose (%)	Strain	Gender	Age (Weeks)		
-	-	-	-	-	-	3	3	C57BL/6	M; F	10	CSS; M; H	[51]
1	10	1	2.5	150	EtOH	-	-	C57BL/6	-	8	CSS; BM; M	[52]
	3–20	-	-	-	-	1–4	1-3	C57BL/6; FVB/Ant and IL-6; BALB/c; A/J; FVB/NJ; B6:129; ICR; SAMP; AKR	M; F	4–12	CSS; BM; M; H	[53,54,55,56,57,58,59,60,61,62,63,64,65,66,67,68,69,70,71,72,73,74,75,76,77,78,79,80,81,82,83,84,85,86,87,88,89,90,91,92,93,94,95,96,97,98,99,100,101,102,103,104,105,106,107,108,109,110,111,112,113,114,115,116,117,118,119,120,121,122,123,124,125,126,127,128,129,130,131,132,133,134]
2	5–12.5	-	-	-	-	2–3	0.5–2	C57BL/6; STAT6; Balb/c	M; F	5–14	CSS; BM; M; H	[135,136,137,138,139]
5	10	-	-	-	-	3	1.70	C57BL/6	F	6	CSS; BM; M	[140]

Legend: CSS: Clinical signs and symptoms (e.g., body weight, mortality, morbidity, stool consistency, number of tumors); BM: Biochemical markers (e.g., TNF-α, TGF-β, IL-6,10,12; IL-1β, IFN-γ, MPO, CD4+ lymphocytes); M: Macroscopic evaluation (e.g., ulcers, thickness, hyperemia, colon weight, and length); H: Histological evaluation (e.g., inflammation); F—Female; M—Male.

## Data Availability

Not applicable.
